# Enzymatic Chicken Pulp Promotes Appetite, Digestive Enzyme Activity, and Growth in *Litopenaeus vannamei*

**DOI:** 10.3390/metabo12080698

**Published:** 2022-07-27

**Authors:** Vivian Hlordzi, Beiping Tan, Xiaohui Dong, Shuang Zhang, Lin Zhu, Ling Zhang, Xiangna Hu, Shuyan Chi

**Affiliations:** 1 Laboratory of Aquatic Nutrition and Feed, College of Fisheries, Guangdong Ocean University, Zhanjiang 524088, China; hlordziviviann@gmail.com (V.H.); bptan@126.com (B.T.); dongxiaohui2003@163.com (X.D.); zhangshuang@163.com (S.Z.); 2 Aquatic Animals Precision Nutrition and High Efficiency Feed Engineering Research Centre of Guangdong Province, Zhanjiang 524088, China; 3 Qingdao Bio-Ways Ingredients Bio-Technology Co., Ltd., Qingdao 266071, China; zhu666lin@163.com; 4 Shenzhen Institute of Quality & Safety Inspection and Research, Shenzhen 518101, China; huxiangna@163.com

**Keywords:** fish soluble pulp, enzymatic chicken pulp, growth performance, appetite, *Litopenaeus vannamei*

## Abstract

Enzymatic chicken pulp (ECP) is an animal protein source that has been proven to be of excellent nutritional content and good quality for the majority of aquatic organisms because of its quality protein, small peptides, palatability, vitamins, and minerals. An 8-week nutritional trial was conducted to assess the effects of an ECP-based diet on the growth performance, digestive enzyme activity, and gene mRNA expression of Pacific white shrimp (*Litopenaeus vannamei*). Fish soluble pulp (FSP) served as the control group while in the experimental groups, and ECPs with three protein contents were used to replace FSP in equal amounts, named ECP1, ECP2, and ECP3, respectively. No significant difference in weight gain rate, specific growth rate, survival rate, or feed conversion ratio was observed (*p* > 0.05) between the groups. Ash content in the Pacific shrimp’s whole body was significantly higher in the ECP1 and ECP3 groups compared to the other groups (*p* < 0.05). Intestinal amylase and protease activities were the highest in the ECP1 and ECP2 groups, respectively (*p* < 0.05). With respect to gene mRNA expression, neuropeptide Y, excitatory amino acid transporter, and fatty acid transport protein 4 were significantly high in the ECP1 group (*p* < 0.05). In conclusion, these three ECPs have their advantages to replace FSP in shrimp feed, but ECP1 is more effective if the effects of digestive enzyme activity, appetite, and expression of growth-related genes are considered.

## 1. Introduction

Penaeidae shrimp farming is rapidly growing, with production exceeding 6 million tons in 2018 and more than 75% of harvest being *Litopenaeus vannamei* [1]. Feed is an essential component of cost-effective aquaculture. Aquaculture’s successes with respect to nutrition and efficiency heavily relies on supplemental meals [2]. Dietary nutritional value is influenced by the quality of the protein sources employed in the production of the meal [3], particularly for aquaculture animals. Fishmeal (FM), which is the major and high-quality protein source in aquafeed, is very difficult to obtain and very expensive due to high demand [4]. It is very important to reduce the amount of FM used in aquafeed by introducing other protein sources.

Significant volumes of waste are produced during the processing of fish and fowl, including internal organs, fat, skin, feet, skeleton, feathers, and blood. The amount of scrap produced is about 43% (*w*/*w*) of the live weight [5]. Fish soluble pulp (FSP) is produced from fish by-products such as skin, scales, bones, swim bladders, roes, intestines, blood, and liver [4]. These by-products contain large amounts of bioactive rich materials which are mostly underutilized, wasted, or discarded. FSP is rich in protein and amino acids which is suitable for the development of the Pacific white shrimp [6]. Since the early 2000s, global fish catch has reduced significantly, therefore reducing the amount of by-products obtained from this sector [1]. This has led to the study of other ingredients as the primary protein source in the diet of aquatic animals [7,8].

Enzymatic chicken pulp (ECP) is among the animal protein sources proven to be of good quality and high nutrition quality in the diet of most aquatic organisms, particularly for carnivorous species [9,10]. Currently, the poultry by-product is primarily used in pet foods because of its quality protein, palatability, essential fatty acids, vitamins, and minerals [11]. ECP is produced from the enzymatic hydrolysis of poultry by-products such as necks, feet, intestines, blood, and undeveloped eggs, exclusive of feathers. These by-products are first ground, heated to kill microorganisms, and later treated with enzymes. Enzymes are thermally inactivated by heating, and centrifuged to obtain two phases, those being supernatant (water and oil) and solid residue [12]. Most pathogenic microorganisms in ECP are destroyed by the rendering process and conditions (time/temperature) used, with a minimum effect on the digestibility of amino acids [13]. Amino acids such as arginine, alanine, and taurine, which improve feed acceptance in crustaceans, have been observed to be slightly higher in ECP compared to poultry by-product meals [14]. ECP is thought to be a proper substitute protein for FM [15,16] in artificial diets for carnivorous and omnivorous aquaculture species due to consistent availability, relatively low price, nutritional composition, and similar nutritional compositions to FM [17,18].

Due to variability in processing specifications such as time, temperature, and enzymes of poultry by-products into ECP, differences in protein, essential amino acid composition, and lipid contents are observed [19]. In modern rendering facilities, advanced processing technologies are used to counter these challenges [20]. As demand for aquafeed is increasing, several authors confirmed that ECP has considerable potential as an ingredient in fish feed production systems [21].

ECPs with different crude protein levels were incorporated into the diets of Pacific white shrimp and compared with an FSP-based diet to study the effect on growth, survival body composition, mRNA gene expression, and intestinal development.

## 2. Materials and Methods

### 2.1. Experimental Diet

In this study, four isonitrogenous (39.7%) and isolipidic (9%) shrimp diets were formulated and prepared. A balanced diet containing FSP was used as a control group (FSP). Three types of ECPs, which contained 32.4%, 33%, and 10.2% crude protein, were added to the diets as ECP1, ECP2, and ECP3 groups, respectively (Table 1 and Table 2). The raw materials were crushed and passed through an 80-mesh sieve. After weighing, all ingredients listed in the formula were mixed thoroughly in a Hobart-type mixer to form a moist dough that was placed into the extruder and shaped into strips. The stripes were air-dried, broken into granules, and sealed in bags until the experiment started.

### 2.2. Experimental Procedure

Healthy Pacific white shrimp larvae were gathered from the southern base of the marine aquaculture seed project of the “863” Program. The purchased shrimps were incubated for two months in an aerated cement pond at the Marine Biological Research Base of Guangdong Ocean University and fed with a commercial diet within this period. Fasting 24 h before the feeding experiment, 720 healthy Pacific white shrimps were batch-weighed to obtain an initial average weight (0.26 ± 0.002 g) and randomly distributed at a stocking density of 30 shrimps per tank in three replicate groups. Indoor fiberglass tanks (0.3 m^3^) were used for this study under a natural photoperiod (12 h light/12 h dark) system. The experimental feed was fed manually four times daily (07:00, 11:00, 17:00, and 21:00) to 10% body weight and later regulated to visual apparent satiation for 8 weeks. Oxygen was provided using single-air stones, water temperature ranged from 28–30 °C, pH 8.0–8.2, dissolved oxygen > 7 mg/L, and salinity 28.5–32, respectively. The feed provided was recorded and about 60% of the water in the tanks was changed daily. Mortalities were weighed and recorded daily.

### 2.3. Sample Collection

80 shrimps were randomly sampled and stored (−20 °C) for the analysis of the initial proximate composition at the onset of the study. 24 h before the cessation of the feeding trial, shrimps were starved. The weight and number of the shrimps per replicate were checked and noted to calculate the survival rate (SR), weight gain rate (WGR), specific growth rate (SGR), and feed conversion ratio (FCR). At random, five shrimps per replicate were selected, weighed, and had their length checked, after which their hepatopancreas and intestines were harvested and weighed to denote their condition factor (CF), viscerosomatic index (VSI), and hepatosomatic index (HSI).

$$\mathrm{Weight}\;\mathrm{gain}\;\mathrm{rate}\;(\%)=100\;\times \;\frac{\left(\mathrm{average}\;\mathrm{final}\;\mathrm{individual}\;\mathrm{weight}\;-\;\mathrm{average}\;\mathrm{initial}\;\mathrm{individual}\;\mathrm{weight}\right)}{\mathrm{average}\;\mathrm{initial}\;\mathrm{individual}\;\mathrm{weight}}$$

$$\mathrm{Specific}\;\mathrm{growth}\;\mathrm{rate}\;(\%/d)=100\;\times \;\frac{\log_{e}\mathrm{average}\;\mathrm{final}\;\mathrm{weight}\;-{\;\log}_{e}\mathrm{average}\;\mathrm{initial}\;\mathrm{weight}}{\mathrm{days}\;\mathrm{of}\;\mathrm{feeding}}$$

$$\mathrm{Feed}\;\mathrm{conversion}\;\mathrm{ratio}=\;\frac{\mathrm{Feed}\;\mathrm{consumed}}{\mathrm{weight}\;\mathrm{gain}}$$

$$\mathrm{Survival}\;\mathrm{rate}\;(\%)=100\;\times \;\frac{\mathrm{Final}\;\mathrm{shrimp}\;\mathrm{number}}{\mathrm{Initial}\;\mathrm{shrimp}\;\mathrm{number}}$$

$$\mathrm{Condition}\;\mathrm{factor}\;(g/{\mathrm{cm}}^{3})=100\;\times \;\left(\frac{\mathrm{final}\;\mathrm{weight}}{{\left(\mathrm{shrimp}\;\mathrm{fork}\;\mathrm{length}\right)}^{3}}\right)$$

(from ref [22])

$$\mathrm{Viscerosomatic}\;\mathrm{index}\;(\%)=100\;\times \;\left(\frac{\;\mathrm{visceral}\;\mathrm{weight}}{\;\mathrm{body}\;\mathrm{weight}}\right)$$

$$\mathrm{Hepatosomatic}\;\mathrm{index}\;(\%)=100\;\times \;\left(\frac{\;\mathrm{hepatopancreas}\;\mathrm{weight}}{\;\mathrm{body}\;\mathrm{weight}}\right)$$

$$\mathrm{Protein}\;\mathrm{efficiency}\;\mathrm{ratio}=\frac{\mathrm{weight}\;\mathrm{gain}}{\mathrm{protein}\;\mathrm{intake}}$$

$$\mathrm{Protein}\;\mathrm{production}\;\mathrm{value}\;(\%)=100\times \frac{\left(\mathrm{Final}\;\mathrm{weight}\;\times \;\mathrm{Crude}\;\mathrm{protein})-(\mathrm{Initial}\;\mathrm{weight}\;\times \;\mathrm{Crude}\;\mathrm{protein}\right)}{\mathrm{Feed}\;\mathrm{given}\;\times \;\mathrm{Crude}\;\mathrm{protein}}$$

#### 2.3.1. Proximate Composition and Analysis

Proximate composition (moisture, crude lipid, crude protein, and ash) of feeds and five shrimps per replicate were analyzed using standard methods obtained from the Association of Official Analytical Chemists [23].

#### 2.3.2. Serum Biochemical Activity

The hemolymph was pooled into 1.5 mL Eppendorf tubes and stored at 4 °C overnight. Stored hemolymph samples were centrifuged (4000 rpm for 10 min at 4 °C) and the serum was harvested for biochemical enzyme activity. Total protein content, aspartate transaminase (AST), alanine transaminase (ALT), catalase (CAT), and lysozyme (LZM) enzyme activities in the serum were evaluated using test kits obtained from Nanjing Jiancheng Institute of Biological Engineering, China. The specific operation methods, absorbance, and calculation formulas were performed according to the test kit instructions. AST and ALT were measured using a full wavelength microplate reader (Thermofisher, Waltham, MA, USA) at 510 nm, and CAT and LZM were measured at 405 nm and 530 nm.

#### 2.3.3. Digestive Enzyme Activity in the Intestine

The intestines of three shrimps per replicate was harvested and frozen in liquid nitrogen and later kept at −80 °C until use. Intestinal amylase, lipase, and protease were determined using the specific operation methods and calculations provided by the commercial test kit (Nanjing Jiancheng Institute of Biological Engineering, Nanjing, China). We weighed the intestine, added 9 times the volume of saline at a ratio of mass (g): volume (mL) of 1:9, homogenized mechanically under ice bath conditions, titled 10% tissue homogenate, centrifuged at 2500 rpm for 10 min, and used the supernatant as the analysis sample. The absorbance of the amylase, lipase, and protease was measured using the microplate reader (Thermofisher, Waltham, MA, USA) at 660 nm, 550 nm, and 680 nm.

#### 2.3.4. Intestinal Microbiota

DNA library sequencing was performed on the Illumina HiseqTM 2500/4000 by Gene Denovo Biotechnology Co., Ltd., (Guangzhou, China). Bioinformatic analysis was performed using Omicsmart, a real-time interactive online platform for data analysis (http://www.omicsmart.com, accessed on 2 February 2022).

#### 2.3.5. Real-Time Quantitative PCR Analysis

Total RNA from the hepatopancreas of three shrimps per replicate was extracted using Trizol (Invitrogen, Waltham, MA, USA) reagent, and the integrity and quality were verified using 1% agarose gel electrophoresis and spectrophotometer (NanoDrop-2000, ThermoScientific, Waltham, MA, USA). Reverse transcription of RNA extracted was performed using a primeScript TM kit (TaKaRa, Dalian, China). A real-time fluorescent quantitative PCR assay was executed to detect the mRNA expression levels for neuropeptide Y (*npy*), fatty acid transport protein 4 (*fatp*4), excitatory amino acid transporter (*eaat*), growth hormone secretagogue receptor type 1 (*gsh-R1*), cholecystokinin receptor type A-like (*cckar*), and cluster of differentiation 36 (*cd*36), with β-actin gene as the housekeeping gene (Bio-Rad CFX96; Bio-Rad Labs, Hercules, CA, USA) (Table 3). Relative gene expression levels were calculated by 2^−^^△△CT^.

### 2.4. Statistical Analysis

Data obtained were analyzed using a one-way analysis of variance (ANOVA). Statistical analyses were performed using the SPSS 22.0 for Windows and general differences were significant at *p* < 0.05. Tukey’s honestly significant difference (HSD) test was used to compare the mean values between individual treatments. Data are represented as mean values of each group of shrimp ± standard error (SE).

## 3. Results

### 3.1. Growth Performance and Survival

The SR of all experimental groups was 80% and above without significant difference (*p* > 0.05), as portrayed in Table 4. No significant difference in IW, WGR, SGR, CF, VSI, or HSI was observed between the experimental groups (*p* > 0.05). FCR was significantly higher in the ECP2 group but was not significantly different from the ECP3 group (*p* < 0.05) (Table 4).

### 3.2. Whole Body Composition

As presented in Table 5, no significant difference in moisture and crude protein was observed (*p* > 0.05). Crude lipid was significantly higher in the FSP and ECP3 groups compared to the ECP1 group (*p* < 0.05), but with no significant difference to the ECP2 group (*p* > 0.05). Ash content was significantly higher in the groups fed ECP diets compared to the FSP group (*p* < 0.05), but no significant difference was observed between the FSP and the ECP2 groups (*p* > 0.05). Whole-body PER was significantly higher in the ECP1 group compared to the ECP3 group (*p* < 0.05) with no significant difference to the FSP and ECP2 groups (*p* > 0.05).

### 3.3. Serum Biochemical Indexes

Serum AST and ALT activities in Table 6 were significantly high in ECP 2 compared to FSP and ECP3 groups (*p* < 0.05) but were not significantly different from the ECP1 group (*p* > 0.05). The serum, CAT, LZM activities, and TP content between the groups portrayed no significant difference (*p* > 0.05).

### 3.4. Digestive Enzyme Activity in Intestine

In the intestine, no significant difference in lipase activity between the groups was observed (*p* > 0.05). Amylase activity in the intestine was significantly higher in the ECP1 group compared to the other groups (*p* < 0.05). Protease activity was significantly high in the ECP1 and ECP2 groups (*p* < 0.05) but was not significantly different from the ECP3 group (*p* > 0.05). (Table 7).

### 3.5. Intestinal Microbiota

#### 3.5.1. Species Composition

**Figure 1:**
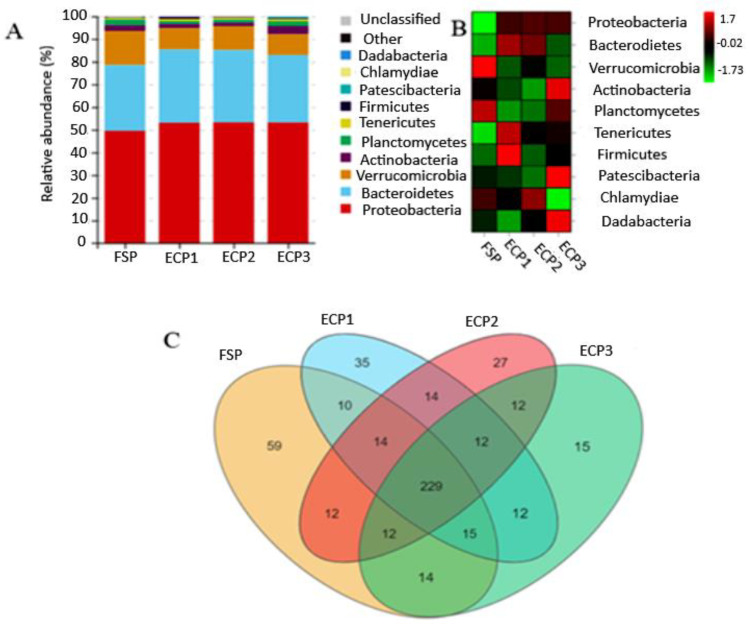
Effect of fish soluble pulp and enzymatic chicken pulp on the bacterial species composition of the intestinal microbiota community of *L. vannamei*. (**A**) Taxonomic distribution; (**B**) Heat map; (**C**) Venn diagram.

In between the groups, most intestinal bacteria observed at the phylum level were classified as Proteobacteria (53%). The 2nd and 3rd predominant phyla were Bacteroidetes (31%) and Perrucomicrobia (10%), respectively. However, the group fed an FSP-based diet had the lowest Proteobacteria level (49.56%), and the highest Verrucomicrobia content at 15.02% (Figure 1A). At the phylum level, the most prevalent bacteria found in the FSP group was Verrucomicrobia. Firmicutes was prevalent in the ECP1 group, Chlamydiae in the ECP2 group, and Patescibacteria in ECP3 as portrayed in the heat map presented in Figure 1B. As displayed in Figure 1C, 229 operational taxonomic units (OTUs) were shared by all the groups. However, the highest unique OTUs were observed in the FSP group (59), followed by the ECP1 group (35), ECP2 group (27), and ECP3 group (15), respectively.

#### 3.5.2. Alpha Diversity

**Figure 2:**
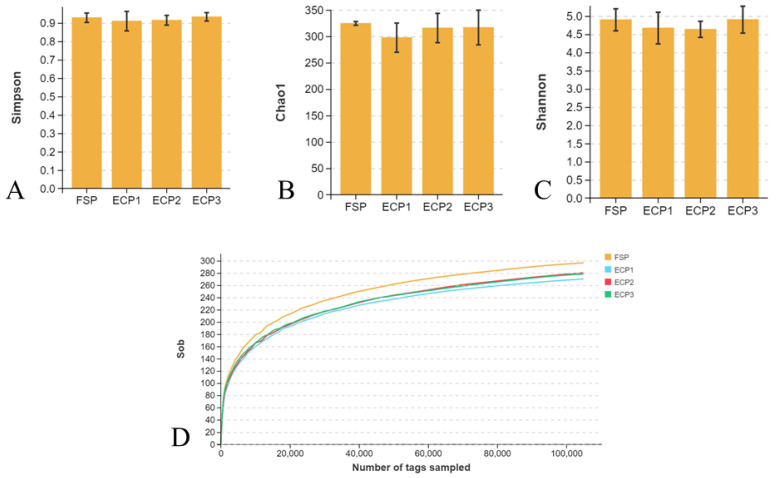
Alpha diversity of present study. (**A**) Simpson diversity; (**B**) Chao1 diversity; (**C**) Shannon diversity; (**D**) Rarefaction.

Alpha diversity results indicated the bacterial community were evenly distributed within the groups. The Simpson, Shannon, and Chao1 diversity were enriched in both FSP and ECP groups and did not differ significantly, as demonstrated in Figure 2A–C. As observed in the rarefaction curve, samples were sequenced at a high depth and near saturation to capture enough variety for all groups (Figure 2D).

#### 3.5.3. Beta Diversity

**Figure 3:**
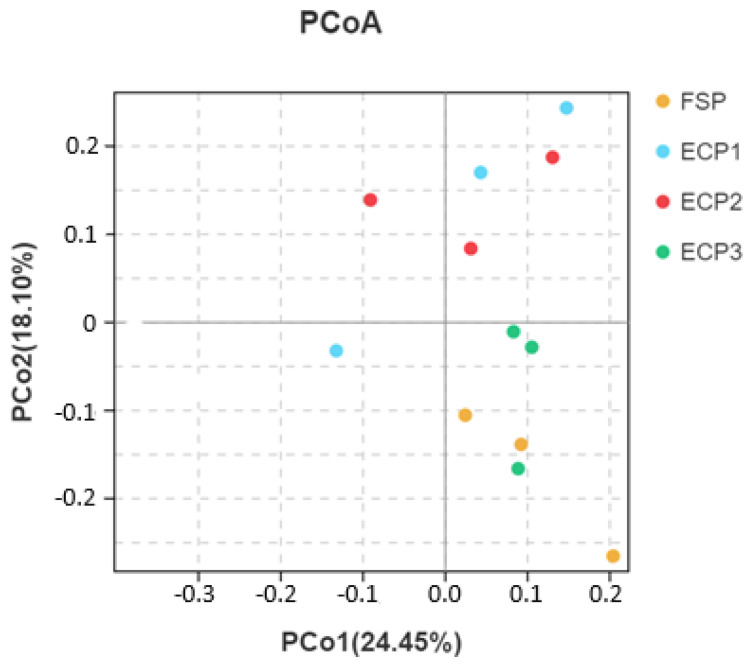
Principal coordinate analysis (PCoA) plot. The scatter plot is of principal coordinate 2 (PCo2) versus principal coordinate 1 (PCo1).

The PCA two-dimensional plot indicated that the gut microbiota of Pacific white shrimp in the FSP group was similar to the ECP3 group, while no association was observed between the FSP group and ECP1 and ECP2 groups (Figure 3).

### 3.6. Gene mRNA Expression in the Hepatopancreas

**Figure 4:**
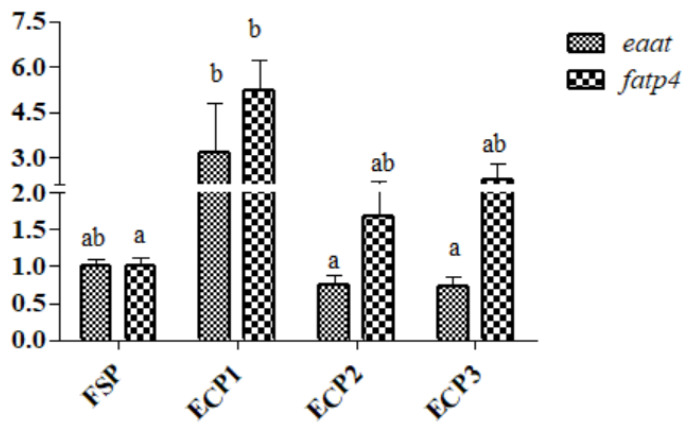
Effect of fish soluble pulp and enzymatic chicken pulp on the expression of *eaat* and *fatp4* on mRNA gene in the hepatopancreas of *L. vannamei*. *eaat-* excitatory amino acid transporter, *fatp4*- fatty acid transport protein 4. Means without superscripts or with the same superscripts do not differ significantly (*p* > 0.05), while those with different superscripts differ significantly (*p* < 0.05).

**Figure 5:**
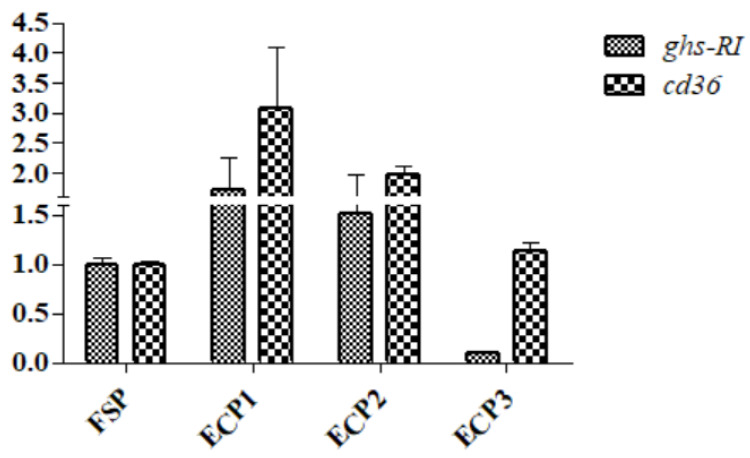
Effect of fish soluble pulp and enzymatic chicken pulp on the expression of *ghs-RI* and *cd36* on mRNA gene in the hepatopancreas of *L. vannamei*. *ghs-RI*—Growth hormone secretagogue receptor type 1, *cd36*—cluster of differentiation 36. Means without superscripts or with the same superscripts do not differ significantly (*p* > 0.05), while those with different superscripts differ significantly (*p* < 0.05).

**Figure 6:**
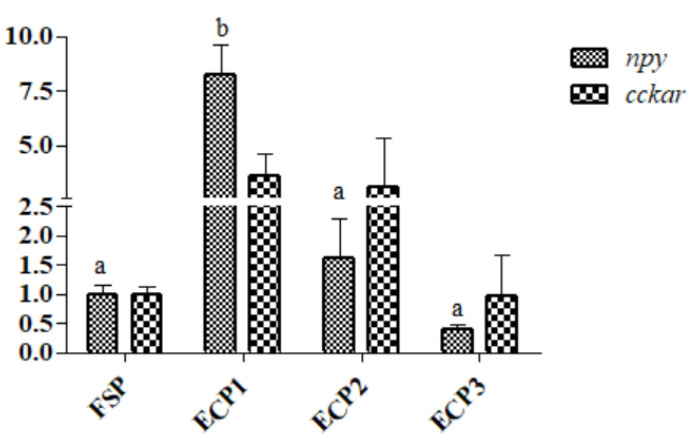
Effect of fish soluble pulp and enzymatic chicken pulp on the expression of *npy* and *cckar* on mRNA gene in the hepatopancreas of *L. vannamei*. *npy*—Neuropeptide Y, *cckar*—cholecystokinin receptor type A-like. Means without superscripts or with the same superscripts do not differ significantly (*p* > 0.05), while those with different superscripts differ significantly (*p* < 0.05).

As demonstrated in Figure 4, the mRNA gene expression of *eaat* was significantly high in the ECP1 group (*p* < 0.05), while *fatp*4 was high in the ECP1 group but was not significantly different from the ECP2 and ECP3 (*p* > 0.05) groups. No significant difference was observed in the gene mRNA expression of *gsh-RI* and *cd*36 (*p* > 0.05) between the groups as demonstrated in Figure 5. The ECP1 group had a significantly high mRNA gene expression of *npy* compared to the other groups (*p* < 0.05), while no significant difference in *cckar* (*p* > 0.05) was observed between the groups (Figure 6).

## 4. Discussion

In this study, no significant difference in WGR and SGR was observed between the groups fed ECP and FSP diets. This might be due to the high digestibility of ECP and FSP and sufficient amino acids (Table 2) [25]. Better digestive enzymatic activities were found in the ECP1 group which has the better growth WGR and PER. Similar results were observed in juvenile rainbow trout (*Oncorhynchus mykiss*) [26], Pacific white shrimp [27], European seabass (*Dicentrarchus labrax*) [28], and tilapia (*Oreochromis niloticus*) [29] fed enzymatic fish by-products, feather-enzymatic hydrolysates, enzymatically hydrolysed aquaculture by-products, and enzymatic poultry by-products, respectively. Poultry and fish by-products have been considered suitable ingredients in the diet of aquatic animals due to their high nutritional characteristics [30]. After enzymatic treatment, these by-products are digested by enzymes and converted from large molecules to small ones; that is, the protein is enzymatically hydrolyzed to small peptides and amino acids. The nutritional value of these ingredients is enhanced [12]. However, this is not in accordance with studies conducted in genetically improved tilapia (*Oreochromis niloticus*) fed enzymatically hydrolyzed chicken liver compared to the non-enzymatically hydrolyzed chicken liver [29]. The composition of ingredients produced from poultry by-products is dependent on the processing method and quality of by-products used [31]. The growth performance of aquatic organisms was improved by a moderate level of enzymatic protein hydrolysate inclusion, whereas growth performance was impaired by a lower or greater inclusion level [32]. Lower WGR observed in genetically improved tilapia could be related to the high enzymatic chicken liver incorporation in their diet [29,33]. Results obtained in this study could be due to a moderate inclusion level of ECP in the diet of Pacific white shrimps.

VSI, HSI, and CF are good representatives of general wellbeing, health, and feed quality in aquatic organisms [34]. Like juvenile rainbow trout (*Oncorhynchus mykiss*) and genetically improved tilapia fed enzymatic fish by-product and enzymatic chicken liver, respectively [26,29], there was no significant difference observed in VSI, HSI, and CF between the groups in this study. Dietary and environmental factors may have contributed to the variations in whole-body composition [31,35]. The whole-body moisture was not influenced by ECP in this study but an increase in the whole-body ash was observed. This was likely due to the high ash content of ECP, which typically comprises bones and feet [31]. However, with reference to the growth performance data, ECP used in the diet of Pacific white shrimps had no negative effect on growth performance, although whole body ash was higher.

In aquatic organisms, the existence and function of molecules, including LZM, CAT, and immunoglobulins, are strongly connected with the immunological functions of immune organs [7,36]. LZM and CAT activities are significant humoral indications of non-specific immunity in aquatic organisms, in addition to key enzymes in the host’s defense system [26,37]. In this study, no significant difference in CAT and LZM was observed in Pacific white shrimps fed diets containing FSP and ECP, but higher levels of CAT and LZM were witnessed in shrimps fed ECP compared to those fed FSP. Similar observations were made by Bae et al. [26] in juvenile rainbow trout fed enzymatically hydrolyzed fish by-products. This could be due to an improvement in the immune component of ECP during enzymatic hydrolysis and the provision of sufficient protein and amino acids in the diets [38]. The total protein level in the serum plays a vital role in the transportation of fatty acids, the maintenance of a steady pH in aquatic organisms [39], and can be used to determine the nutritional status of an organism [40]. There was no significant difference in total protein content of Pacific white shrimps between the groups which was also observed in juvenile rainbow trout [26].

The gut microbiota plays a variety of important roles in the host, notably immune system modification, pathogen defense, anaerobic peptide, and protein metabolism, in addition to the processing of non-digestible food fibers [41,42]. Although no significant difference was observed, Pacific white shrimps fed FSP diets had higher OTUs and alpha diversity indices (Simpson, Chao1, Shannon, and Rarefaction) compared to those fed ECP diets. The nutrient quality in the diet and rearing environment may influence the abundance of Proteobacteria, Bacteroidetes, and Actinobacteria in the gut of Pacific white shrimps [43]. Proteobacteria and Bacteroidetes were the most abundant bacteria found in the gut of the Pacific white shrimps in the groups. Proteobacteria, Bacteroidetes, and Actinobacteria were observed to be higher in Pacific white shrimps fed ECP diets compared to those fed FSP. Proteobacteria are the most common phylum of bacteria in the aquatic systems and aquatic animals’ intestines [44]. Bacteroidetes helped to improve the digestion and absorption of nutrients in the feed of Pacific white shrimp [44,45], and in this experiment, more abundant Bacteroidetes were observed in the ECP1 group compared to other groups, which in turn increased WGR, SGR, and CF. Actinobacteria, although a small bacterial group, plays an important part in the formation and maintenance of the immune system and feed metabolism [46]. The Pacific white shrimps fed ECP-based diets had a relatively high Actinobacteria composition and had a higher LZM content in the intestine of Pacific white shrimp. This suggests that ECP could help Pacific white shrimps’ immunological responses.

The transport of nutrients is very important for animal health. Amino acid transporter expression is a key measure of aquatic species’ ability to absorb amino acids [47]. They are primarily responsible for the transmembrane transport of amino acids in the body, in addition to promoting amino acid assimilation, protein synthesis, and maintaining physiological functioning [48,49]. In this study, ECP1 had a higher *eaat* and *fatp4* mRNA expression level compared to the other groups. Results in this study were similar to results obtained by Zhuang et al. [14] who observed a higher expression level of b^0^ neutral amino acid transporter 1 (*b*^0^*at1*) and neutral and cationic amino acid transporter (*y^+^lat2*) in juvenile turbot fed enzymatic hydrolysis of chicken by-product in high plant-based protein. This could be due to the appropriate amount of free amino acids in ECP1, leading to the stimulation of the transporters of amino acids located in the hepatopancreas [50], which is conducive for growth [51,52].

**Figure 7:**
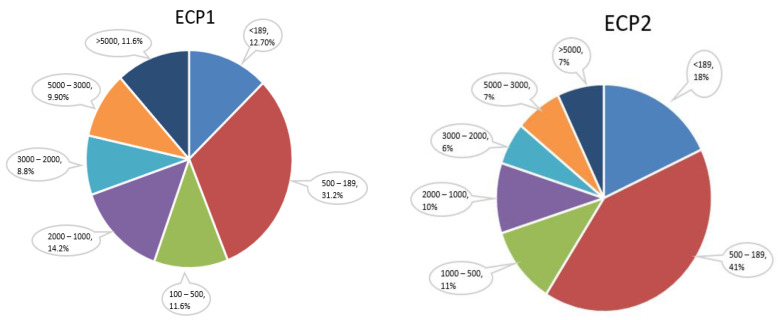
Distribution of molecular weight of small peptides in the raw ingredients of ECP1 and ECP2.

Glycine, alanine, and proline have strong predatory effects on aquatic animals [53]. The three amino acid contents in the ingredients of FSP, ECP1, ECP2, and ECP3 used in this experiment were 6.68%, 8.13%, 8.28%, and 1.47%, respectively (Table 2). According to the three amino acid content, the appetizing effects of the ECP1 and ECP2 treatment should be better. The gene *npy* expression was upregulated in these two groups, although the expression level in ECP2 was without significant difference compared to the FSP. Another reason could be the different small peptide contents in ECP1 and ECP2 (Figure 7) making the ECP1 feed more attractive, thus significantly increasing the expression of *npy* mRNA level of the shrimps [54].

## 5. Conclusions

ECP1, ECP2, and ECP3 with different protein contents, amino acids, and small peptides, although obtained from the same raw material with different processes, demonstrated different effects. Shrimp that ingested a diet with ECP1 indicated better growth performance, digestive enzyme activity, and upregulated mRNA expression of appetite and growth related genes compared to the other groups. In ECP2, serum AST and ALT were significantly higher than in the other groups. ECP1, which contains a 32.4% crude protein level, is recommended for the diet of Pacific white shrimp larvae.

## Figures and Tables

**Table 1:** Formulation and proximate composition of the experimental diets (% dry matter).

Ingredients	FSP	ECP1	ECP2	ECP3
Brown fish meal	20	20	20	20
Fish soluble pulp	4	0.00	0.00	0.00
ECP 1	0	4	0	0
ECP 2	0	0	4	0
ECP 3	0	0	0	4
Soybean meal	15	15	15	15
Cottonseed protein	11	11	11	12.5
Wheat flour	24	24	24	24
Peanut meal	14	14	14	14
Shrimp shell meal	3	3	3	3
Fish oil	1.6	1.6	1.6	1.6
Soy lecithin	1.5	1.5	1.5	1.5
Soybean oil	1.5	1.5	1.5	1.3
Calcium monophosphate	1	1	1	1
Premix (Vitamin + Mineral)	1	1	1	1
Cholesterol	0.15	0.15	0.15	0.15
Antioxidant	0.45	0.45	0.45	0.45
Microcrystalline cellulose	1.3	1.3	1.3	0
Choline Chloride	0.4	0.4	0.4	0.4
Attractant	0.1	0.1	0.1	0.1
Total %	100	100	100	100
Nutrient composition (%)				
Moisture	9.34	9.55	9.32	9.21
Crude protein (dry matter)	38.34	38.09	38.29	38.13
Crude lipid (dry matter)	7.14	6.77	6.79	6.55
Ash (dry matter)	10.22	10.24	10.33	10.11

**Table 2:** Amino acid composition (%).

	FSP	ECP1	ECP2	ECP3	Cottonseed Protein
Aspartic acid	1.99	1.79	1.82	1.25	5.68
Glutamic acid	2.4	2.43	2.48	1.48	12.33
Serine	0.72	0.73	0.74	0.17	2.48
Histidine	1.64	0.82	0.84	0.16	1.73
Arginine	2.35	2.05	2.09	0.52	7.75
Threonine	0.98	0.62	0.63	0.14	1.93
Glycine	1.78	3.28	3.34	0.35	2.45
Alanine	2.94	1.66	1.69	0.71	2.29
Proline	1.96	3.19	3.25	0.41	2.14
Tyrosine	0.78	0.41	0.42	0.21	1.71
Valine	1.04	0.66	0.67	0.29	2.75
Methionine	0.46	0.4	0.41	0.12	0.76
Cystine	0.2	0.39	0.40	0.03	0.76
Isoleucine	0.91	0.67	0.68	0.28	1.87
Leucine	1.72	1.32	1.35	0.29	3.22
Phenylalanine	1.2	0.84	0.86	0.15	3.39
Lysine	2.05	1.23	1.25	0.34	2.62

**Table 3:** Primer sequence used for real-time quantitative PCR analysis.

Gene	Primer	Source
*npy*	F: GGTGATGTCGAAGTGGCCGGAGTTG R: ACCTCGCCAGGGAGAAGCGGAACCA	GFRP01055388
*fatp4*	F:CCGACGGGCAAAGCGACTGAACCA R: TCTATTCCACCAGGTATCTTTATCG	KY271629
*eaat*	F: GTTACAACATCAAACCCGAGACAG R:CCCGAGAAGGTGAAGATGAGGAGC	GGUK01021174
*ghs-RI*	F: TGCGAAGGAGGAACTCTGAACATT R: CCAAGTAAGTCGCTTCCTGGCTCT	HAAW01018270
*cckar*	F: ATCGTGTCCCTTGTGCTGTCTGTT R: GTCATCGCCGTCATCTTCTTCGTC	XM_027379811
*cd36*	F: AACCAAGGTCCTGACCATCAC R: AGGTGAGAGTCGACGAGGAA	[24]
*β-actin*	F: AAGATGTGTGACGACGAAGTAGC R: AGGATACCTCGCTTGCTCTG	GFRP01025709

*npy*—neuropeptide Y, *fatp4*—fatty acid transport protein 4, *eaat*—excitatory amino acid transporter, *ghs-RI*—growth hormone secretagogue receptor type 1, *cckar*—cholecystokinin receptor type A-like, *cd36*—cluster of differentiation 36, *β-actin*—beta-actin.

**Table 4:** Effect of fish soluble pulp meal and enzymatic chicken pulp on the growth and survival of *L. vannamei*.

	FSP	ECP1	ECP2	ECP3
IW	0.26 ± 0.00	0.26 ± 0.00	0.26 ± 0.01	0.26 ± 0.00
WGR/%	2789.92 ± 34.42	2923.21 ± 17.57	2818.48 ± 57.17	2553.41 ± 46.36
SGR (%/d)	5.53 ± 0.08	5.87 ± 0.06	5.52 ± 0.17	5.49 ± 0.22
SR,%	80.00 ± 3.85	88.33.00 ± 2.15	82.22 ± 5.55	86.67 ± 6.67
FCR	1.64 ± 0.02 ^a^	1.58 ± 0.03 ^a^	1.84 ± 0.04 ^b^	1.70 ± 0.5 ^ab^
CF, g/cm^3^	0.81 ± 0.03	0.87 ± 0.01	0.81 ± 0.03	0.81 ± 0.01
VSI,%	5.85 ± 0.09	5.55 ± 0.22	5.11 ± 0.34	5.07 ± 0.32
HSI,%	4.65 ± 0.12	4.11 ± 0.20	4.00 ± 0.28	3.96 ± 0.14

Note: Values are mean values of each group of shrimp (3 replicates) ± SE. Means in each row without superscripts or with the same superscripts do not differ significantly (*p* > 0.05), while those with different superscripts differ significantly (*p* < 0.05). IW-Initial weight, WGR%-Weight gain rate, SGR%/d—Specific growth rate per day, SR%—Survival rate, FCR—Feed conversion ratio, CF%—Condition factor, VSI%—Viscerosomatic index, HSI-Hepatosomatic index.

**Table 5:** Effect of fish soluble pulp and enzymatic chicken pulp on the whole-body composition of *L. vannamei*.

	FSP	ECP1	ECP2	ECP3
Moisture	73.44 ± 0.26	74.59 ± 0.57	73.62 ± 0.19	74.05 ± 0.21
Crude protein	69.28 ± 2.11	63.76 ± 3.13	69.23 ± 2.08	64.39 ± 0.63
Crude lipid	6.83 ± 0.78 ^b^	5.74 ± 0.16 ^a^	6.41 ± 0.16 ^ab^	7.47 ± 0.40 ^b^
Ash	10.43 ± 0.01 ^a^	11.75 ± 0.04 ^b^	10.98 ± 0.35 ^ab^	11.81 ± 0.25 ^b^
PER	4.67 ± 0.80 ^ab^	5.41 ± 0.11 ^b^	4.62 ± 0.10 ^ab^	4.48 ± 0.27 ^a^
PPV	40.17 ± 2.09	40.64 ± 6.83	39.57 ± 2.34	32.08 ± 1.00

Note: Values are mean values of each group of shrimps (three replicates) ± SE. Means in each row without superscripts or with the same superscripts do not differ significantly (*p* > 0.05), whiles those with different superscripts differ significantly (*p* < 0.05). PER—Protein efficiency rate, PPV—protein production value.

**Table 6:** Effect of fish soluble pulp and enzymatic chicken pulp in the serum enzyme activities of *L. vannamei*.

	AST, U/L	ALT, U/L	LZM, U/ml	CAT, U/ml	TP, g/ml
FSP	17.94 ± 3.58 ^a^	46.76 ± 4.17 ^ab^	275.00 ± 21.43	7.08 ± 3.23	299.67 ± 8.51
ECP1	26.72 ± 4.20 ^ab^	58.42 ± 4.19 ^bc^	285.71 ± 19.88	13.28 ± 3.87	297.79 ± 20.72
ECP2	41.76 ± 0.63 ^b^	59.38 ± 0.37 ^c^	291.67 ± 5.19	15.86 ± 6.88	324.81 ± 7.92
ECP3	12.07 ± 2.93 ^a^	40.55 ± 1.12 ^a^	300.00 ± 5.46	10.45 ± 1.18	289.89 ± 13.86

Note: Values are mean values of each group of shrimps (three replicates) ± SE. Means in each row without superscripts or with the same superscripts do not differ significantly (*p* > 0.05), while those with different superscripts differ significantly (*p* < 0.05).

**Table 7:** Effect of fish soluble pulp and enzymatic chicken pulp in the intestinal digestive enzyme activities of *L. vannamei*.

	Lipase, U/gprot	Amylase, U/mgprot	Protease, U/mL
FSP	0.81 ± 0.26	3.07 ± 0.27 ^a^	1352.24 ± 116.5 ^a^
ECP1	1.06 ± 0.05	5.47 ± 0.03 ^b^	1944.10 ± 156.08 ^b^
ECP2	0.52 ± 0.04	3.05 ± 0.03 ^a^	2322.53 ± 53.75 ^b^
ECP3	0.60 ± 0.01	3.16 ± 0.01 ^a^	1828.34 ± 124.36 ^ab^

Note: Values are mean values of each group of shrimps (three replicates) ± SE. Means in each row without superscripts or with the same superscripts do not differ significantly (*p* > 0.05), while those with different superscripts differ significantly (*p* < 0.05).

## Data Availability

The datasets generated and/or analyzed during the current study are available from the corresponding author on reasonable request.
